# Plasma activity of Thioredoxin Reductase as a Novel Biomarker in Gastric Cancer

**DOI:** 10.1038/s41598-019-55641-6

**Published:** 2019-12-13

**Authors:** Wei Peng, Zhaofei Zhou, Yuejiao Zhong, Yan Sun, Yajing Wang, Zili Zhu, Wenxuan Jiao, Man Bai, Jing Sun, Hanwei Yin, Jianwei Lu

**Affiliations:** 1 Department of Medicine, Jiangsu Cancer Hospital & Jiangsu Institute of Cancer Research & The Affiliated Cancer Hospital of Nanjing Medical University, Nanjing, China; 2 Nantong Tumor Hospital, Nantong, China; 3 State Key Laboratory of Natural and Biomimetic Drugs, Peking University Health Science Center, Beijing, China; 4 Keaise Center for Clinical Laboratory, Wuhan, China

**Keywords:** Diagnostic markers, Biomarkers, Cancer, Tumour biomarkers, Gastrointestinal cancer

## Abstract

Gastric cancer (GC) is one of the leading malignancies around the world. Identification of novel and efficient biomarkers for GC diagnosis and evaluation of therapeutic efficiency could improve the therapeutic strategy in future clinical application. This study aims to evaluate the levels of plasma thioredoxin reductase (TrxR) activity in GC patients to confirm its validity and efficacy in GC diagnosis and evaluation of therapeutic efficiency. 923 cases were enrolled in the current study. In the group of GC patients before clinical intervention, plasma TrxR activity [9.09 (7.96, 10.45) U/mL] was significantly higher than in healthy controls [3.69 (2.38, 5.32) U/mL]. The threshold of TrxR activity for GC diagnosis was set at 7.34 U/mL with a sensitivity of 85.5% and a specificity of 97.9%. In GC patients after chemotherapy, plasma TrxR activity was remarkably higher in patients with progressive disease or uncontrolled condition [10.07 (8.19, 11.02) U/mL] compared with patients with complete or partial response [7.12 (6.08, 8.37) U/mL] in response to chemotherapy. TrxR activity displayed the higher efficiency to distinguish between GC patients with two distinct clinical outcomes than carcinoembryonic antigen (CEA), cancer antigen 72-4 (CA72-4) and cancer antigen 19-9 (CA19-9). Moreover, combination of TrxR, CEA, CA72-4 and CA19-9 was demonstrated to be more effective in both GC diagnosis and evaluation of therapeutic efficiency than was each biomarker individually. Together, plasma TrxR activity was identified as a novel and efficient biomarker of GC, both in diagnosis and monitoring of therapeutic efficiency in response to chemotherapy.

## Introduction

Gastric cancer (GC) is the fifth most common cancer worldwide, the fourth most commonly occurring cancer in men and the seventh most commonly occurring cancer in women^1^. There were over 1 million new cases in 2018, and the highest incidence rates of countries are mostly located in Asia including South Korea, Mongolia, Japan and China^1,2^. In China, GC rank the second highest tumor incidence rates (10.26%) and the third highest tumor mortality rates (10.74%)^3–5^. Approximately 85% of GC cases are diagnosed as adenocarcinoma based on histologic classification. Compared with other carcinomas, GC carries a far worse prognosis and high recurrence (around 30%); while the overall 5-year survival rate after diagnosis remains around 27%^6,7^. Early clinical interventions are essential to improve the 5-year survival rates and reduce GC recurrence, and largely depend on the early and accurate diagnosis of GC. However, delays and omission commonly occur in the clinical diagnosis of GC.

Currently, invasive diagnostic strategies such as gastroscope are widely applied in the clinical diagnosis of GC. However, these tests usually fail to uncover the hidden or subclinical lesions and lead to high false-positive rates especially in early-stage GC^8^. Additionally, pain and inconvenience led by gastroscope and needle biopsy always make it unfeasible for some patients. Recently, several tumor-specific proteins have been identified as GC biomarkers in the clinical diagnosis, such as carcinoembryonic antigen (CEA), cancer antigen 72-4 (CA72-4) and cancer antigen 19-9 (CA19-9)^9–11^. However, the appliance of these biomarkers has limited application owing to their low sensitivity not only in the diagnosis of early-stage GC, but also in the monitoring of GC therapeutic efficiencies in response to chemotherapy^12,13^. Therefore, it is a top priority to identify a novel GC biomarker with high sensitivity and specificity to improve the clinical GC diagnosis.

Mammalian thioredoxin reductase (TrxR) is a selenium-containing oxidoreductase that is responsible for catalyzing the NADPH-dependent reduction reaction of thioredoxin (Trx) disulfide^14–20^. TrxR is also known to participate in several redox-sensitive signaling cascades that mediate numerous physiological processes, especially cell survival, proliferation, growth and apoptosis^20–32^. TrxR1, the major isoform of TrxR in the cytoplasm, has been observed to be highly expressed in various malignancies including non-small cell lung carcinoma (NSCLC), breast cancer and hepatocellular carcinoma^33–35^. Previous studies have suggested TrxR as a potential and effective clinical biomarker of early-stage diagnosis and prognosis after chemotherapy in NSCLC, breast cancer, prostate cancer and liver cancer^35–38^. Moreover, TrxR was also reported to be overexpressed in BGC823 GC cell line, and the inhibition of TrxR activity by the TrxR-specific inhibitor resulted in a robust antitumor effect in BGC823 cell line as well as *in vivo* in GC xenograft mice, suggesting that TrxR may be a potential biomarker involved in the GC diagnosis and evaluation of therapeutic efficiency^39,40^.

Currently, we conducted a retrospective study aiming to analyze the efficiency of TrxR activity as a plasma biomarker in the GC diagnosis and evaluation of therapeutic efficiency. Furthermore, we compared the levels of TrxR activity with CEA, CA72-4, CA19-9 and combinations thereof in a large clinical population. Our results revealed that TrxR could be a valuable biomarker of GC diagnosis and therapeutic evaluation for future clinical application.

## Materials and Methods

### Patients

Patients with pathologically diagnosed gastric cancer and sex- and age-matched healthy controls, as shown in Supplemental Table S1, were continuously recruited from Jiangsu Cancer Hospital (Jiangsu, China), from 2017 to 2019. All healthy controls were in normal conditions based on their complete blood test, liver/kidney functions and chest X-ray examination. Gastric cancer was defined based on computed tomography (CT) results and confirmed by histopathology according to the World Health Organization Classification of Tumors^41^. Tumor stage was defined according to the 8^th^ IASLC/AJCC staging system^42^.

### Specimen characteristics

Sample collection was performed as described in previous publication^38^. EDTA or anticoagulant-free tubes were used to collect 5 mL samples of preoperative peripheral blood. Samples were then centrifuged at 3,500 rpm at room temperature for 5 minutes within 2 hours of collection. Supernatants were collected immediately to measure the levels of GC biomarkers.

### Analysis of tumor markers

Levels of GC-related tumor markers CEA, CA19-9 and CA72-4 in patients were measured at the indicated time of their visit to the hospital. As described in previous literature^12,38^, CEA, CA19-9 and CA72-4 were analyzed based on electrochemiluminescence immunoassay (ECLIA) using Cobas analyzer (Roche Diagnostics, Mannheim, Germany), and performed according to the manufacturer’s instruction. According to the standard clinical guideline from Chinese society of clinical oncology (CSCO), the cut-off value for the tumor markers were set at 3.5 ng/mL for CEA, 39 U/mL for CA19-9, and 6.9 U/mL for CA72-4^43,44^.

### Analysis of TrxR activity

According to the previous publication^33,36–38^, 5, 5′-dithiobis (2-nitrobenzoic) acid (DTNB) reduction assay was widely applied to measure the TrxR activity *in vitro*, especially in biological samples. Basically, TrxR activity was measured by the enzymatic activity of TrxR to catalyze the reduction of DTNB with NADPH to 5-thio-2-nitrobenzoic acid (TNB2-), which generates a strong yellow color with maximum absorbance at 412 nm^45–47^. In the current study, a specific TrxR inhibitor is used to deduct the background in plasma and determine the reduction of DTNB due only to TrxR activity. TrxR activity was measured by commercially available colorimetric assay kits (Clairvoyance Health Technology, China), and performed according to the manufacturer’s instruction^36–38^. Full details are provided in Supplemental experimental procedures.

### Immunohistochemical analysis

The immunohistochemical analysis of normal gastric mucosa and adenocarcinoma tissues was performed as described previously^48,49^. Briefly, the tissues were dewaxed in xylene (twice for 5 min) and rehydrated in a series of ethanol solutions. Antigen retrieval was performed in sodium citrate buffer in a boiling water bath for 10 min. After cooling, three times washing with PBS (5 min each time) and blocking using a hydrogen peroxide solution, the tissue sections were incubated with the primary antibody (1:500) to TrxR1 (Abcam, USA, 16840) for 8 hours. Secondary anti-rabbit IgG (Maxim, China) was then used to incubate for 30 min^48^. Staining was performed using 3,3′-diaminobenzidine. TrxR expression was scored based on the area and staining intensity according to the previous literature^48^.

### Statistical analysis

The value of TrxR activity and other tumor biomarkers in human samples did follow the skewed distribution instead of the normal distribution; and therefore, results are described as percentages for categorical variables and as medians (interquartile ranges, IQRs) for the continuous variables. As suggested by previous publications^36,38^, Chi-squared test were used to calculate and compare the proportions, while non-parametric Mann-Whitney test were applied to compare the continuous variables between groups with a Bonferroni correction. The diagnostic efficacy of biomarkers was evaluated based on the receiver operating characteristic (ROC) curves, the area under the curve (AUC) values and the 95% confidence interval (CI). Logistic regression analysis was used to evaluate the correlations between plasma TrxR activity and other tumor biomarkers by the Spearman test^38^. All statistical analyses were performed using GraphPad Prism 7 (version 7.0; Graphpad, La Jolla, CA, USA) and SPSS version 19.0 (SPSS Inc., Chicago, IL, USA). *P* values were calculated by using the non-parametric Mann-Whitney U-test. *P* values less than 0.05 were considered statistically significant.

### Ethics statement

This study was approved by the Ethics Committee of Jiangsu Cancer Hospital (Jiangsu, China). The methods were applied in accordance with the approved guidelines. Informed consent was obtained from all patients.

## Results

A total of 923 specimens were recruited in the current study, including 131 specimens from patients with GC before clinical intervention, 662 specimens from patients with GC after chemical drug treatment, and 130 healthy controls.

### Plasma TrxR activity in patients with gastric cancer and healthy controls before clinical intervention

**Figure 1:**
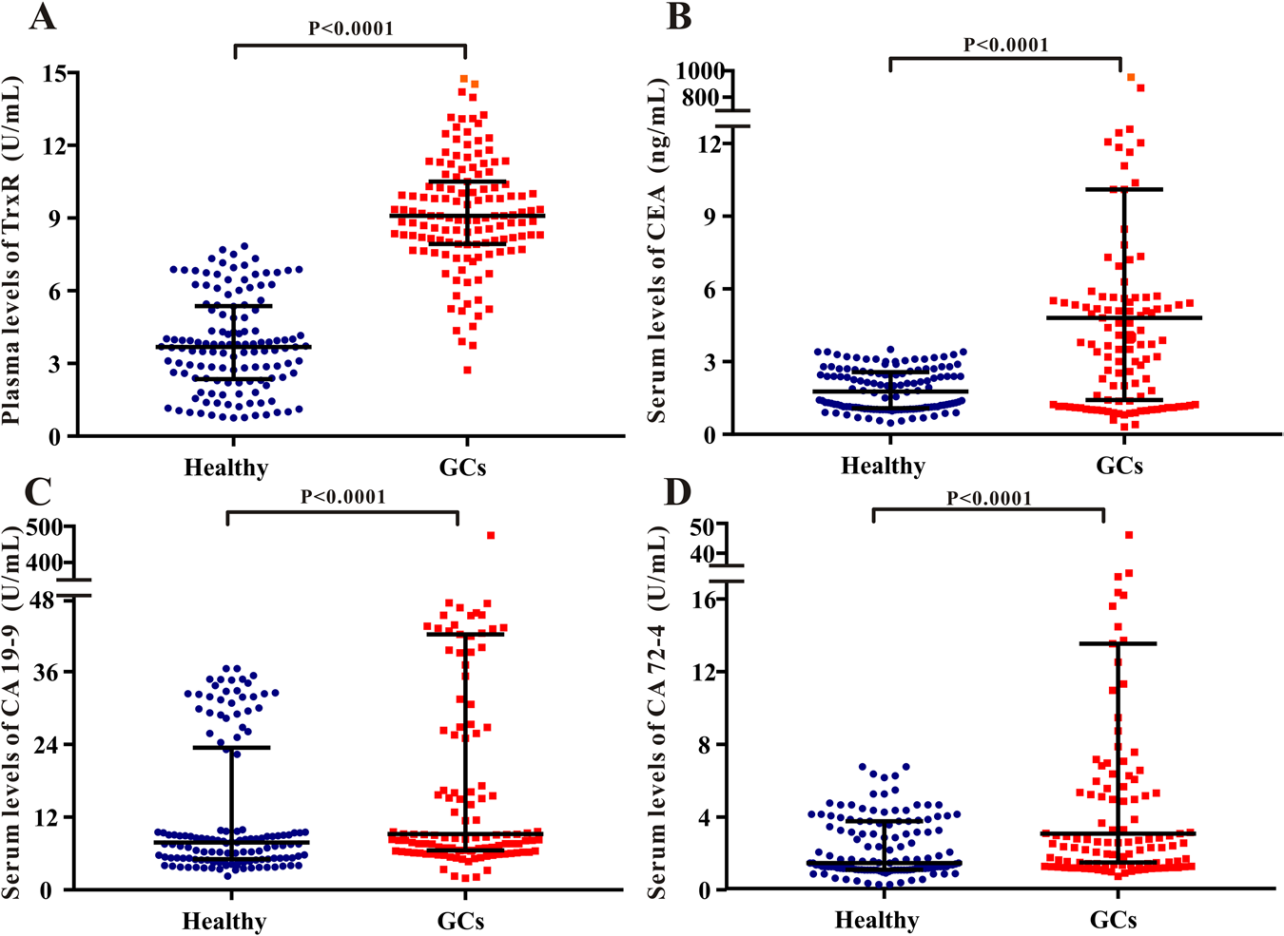
Scatter plot of the distribution of plasma TrxR (**A**), serum CEA (**B**), serum CA19-9 (**C**), and serum CA72-4 (**D**) levels in GC patients and healthy controls. The black horizontal lines are median values and interquartile ranges. P values were determined by the Mann–Whitney U test.

The levels of plasma TrxR activity, serum CEA, serum CA19-9, and serum CA72-4 [median (IQR)] in patients with GC and healthy controls before clinical interventions were detected and analyzed. As shown in Fig. 1A, the plasma levels of TrxR activity in patients with GC [9.09 (7.96, 10.45) U/mL] were significantly higher (P < 0.0001, Mann-Whitney U test) than that in healthy controls [3.69 (2.38, 5.32) U/mL]. Similarly, levels of serum CEA, CA19-9, and CA72-4 in patients with GC were also significantly higher relative to healthy controls, suggesting plasma TrxR activity, as well as CEA, CA19-9, and CA72-4 are sensitive biomarkers elevated in GC before clinical intervention (Fig. 1B—D). Meanwhile, these findings were further strengthened by the overexpression of TrxR protein in GC tissues in comparison with normal gastric mucosa tissues. As shown in Supplemental Fig. S1, TrxR expression was determined to be positive in 60% of the GC tissues. However, its expression in normal gastric mucosa tissue was rarely seen (3/20).

### The efficacy of TrxR activity as a diagnostic biomarker of gastric cancer

**Figure 2:**
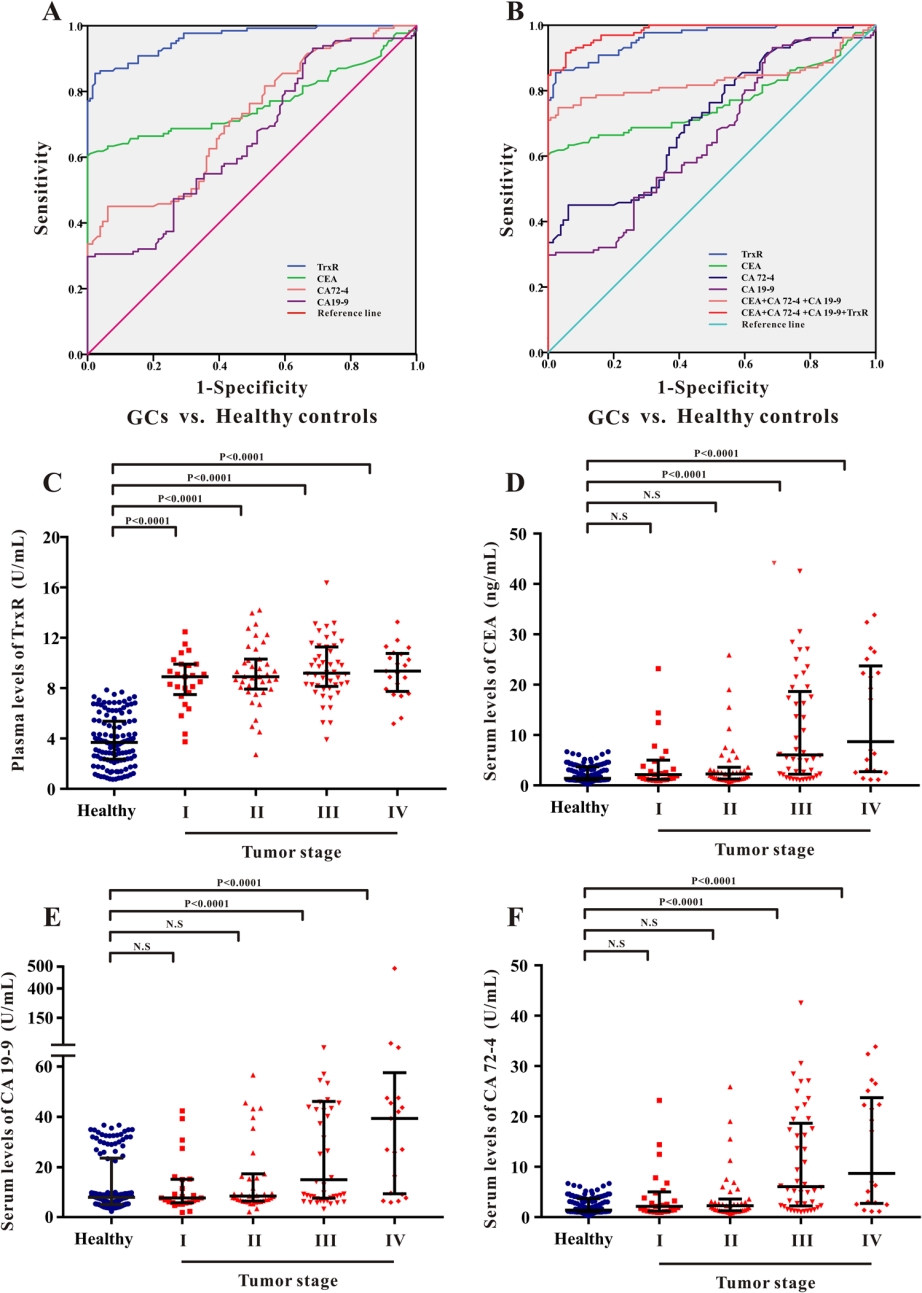
(**A,B**) ROC curve analyses of TrxR, CEA, CA19-9, CA72-4 (**A**), and the combinations thereof (**B**) for the differentiation of GCs and healthy controls. (**C–F**) Scatter plot of the distribution of plasma TrxR (**C**), serum CEA (**D**), serum CA19-9 (**E**), and serum CA72-4 (**F**) levels in GC patients with various pathological TNM stages. The black horizontal lines are median values and interquartile ranges. P values were determined by the Mann–Whitney U test. N.S: no statistical significance.

ROC curve analysis was performed to evaluate the efficacy of TrxR plasma activity as a diagnostic biomarker for GC. The maximal Youden Index (sensitivity + specificity-1) was used to calculated the optimal cut-off value of TrxR activity to distinguish between GC patients with healthy controls. As shown in Fig. 2A and Table 1, the critical value of TrxR activity for GC diagnosis was set at 7.34 U/mL based on the ROC curve (AUC 0.963; 95% CI, 0.943-0.983) with a sensitivity of 85.50% and a specificity of 97.69%. As a comparison, CEA displayed the second highest AUC in differentiating GC patients from healthy controls (Fig. 2A and Table 1; 0.764; 95% CI, 0.701–0.826). Meanwhile, CA19-9 and CA72-4 also exhibited moderate capacities for differentiating GC patients from healthy controls with AUCs of 0.657(95% CI, 0.591–0.723) and 0.719 (95% CI, 0.658–0.780), respectively. However, the sensitivities of both CA19-9 and CA72-4 were blow 50%, suggesting the high risk of false-negative rate using CA19-9 and CA72-4 in GC diagnosis. These results indicated that the diagnostic efficacy of plasma TrxR activity in GC was greater than that of CEA, CA19-9 and CA72-4.

**Table 1:** The diagnostic efficiency of TrxR, CEA, CA72-4, CA19-9 and combinations thereof in differentiating between GC patients and healthy controls.

Tumor markers	GC patients before clinical intervention						
	AUC (95%CI)	SEN%	SPE%	PPV%	NPV%	PLR	NLR
**GC patients vs. healthy controls**							
TrxR	0.963 (0.943–0.983)	85.50	97.69	97.37	87.07	37.05	6.74
CEA	0.764 (0.701–0.826)	61.07	99.23	98.76	71.82	79.39	2.55
CA19–9	0.657 (0.591–0.723)	29.77	100.00	100.00	58.74	−	1.42
CA72-4	0.719 (0.658–0.780)	45.04	93.85	87.98	63.07	7.32	1.71
CEA + CA19-9 + CA72-4	0.834 (0.778–0.891)	78.41	96.92	96.05	79.37	24.31	3.85
CEA + CA19-9 + CA72-4 + TrxR	0.982 (0.970–0.993)	91.60	94.62	94.45	91.85	17.01	11.27

Abbreviations: SEN: sensitivity; SPE: specificity; PPV: positive predictive value; NPV: negative predictive value; PLR: positive likelihood ratio; NLR: negative likelihood ratio.

The diagnostic cut-off value of TrxR activity levels was 7.34 U/mL

Furthermore, a binary logistic regression analysis was applied to explore the diagnostic efficacy of the combination of these tumor biomarkers in GC diagnosis. As shown in Fig. 2B and Table 2, the combination panel of CEA, CA19-9 and CA72-4 exhibited an improved diagnostic efficacy for GCs (AUC 0.834; 95% CI, 0.778-0.891) relative to any individual biomarker (P < 0.05). Notably, when adding TrxR into this combination panel, diagnostic efficiency for GC was further elevated (AUC 0.982; 95% CI, 0.970–0.993) relative to the combination of only CEA, CA19-9 and CA72-4 (P < 0.01). These results offered a novel diagnostic panel of 4 biomarkers (TrxR, CEA, CA19-9, CA72-4) in GC diagnosis for future clinical application.

**Table 2:** The diagnostic efficiency of CEA, CA72-4, CA19-9, and TrxR in discriminating GC patients with various pathological TNM stages from healthy controls.

Tumor markers	GC patients before clinical intervention						
	AUC (95%CI)	SEN%	SPE%	PPV%	NPV%	PLR	NLR
**GCs stage I vs. healthy controls**							
TrxR	0.948 (0.900–0.995)	80.00	97.69	97.20	83.01	34.67	4.88
CEA	0.518 (0.363–0.673)	32.00	93.85	83.87	57.98	5.20	1.38
CA72-4	0.613 (0.491–0.735)	28.00	93.85	81.98	56.59	4.55	1.30
CA19-9	0.493 (0.375–0.612)	84.00	26.15	53.22	62.04	1.14	1.63
**GCs stage II vs. healthy controls**							
TrxR	0.955 (0.914–0.997)	84.62	97.69	97.35	86.39	36.67	6.35
CEA	0.653 (0.530–0.775)	48.72	99.23	98.45	65.93	63.33	1.94
CA72-4	0.605 (0.508–0.703)	79.49	43.08	58.27	67.74	1.40	2.10
CA19-9	0.593 (0.494–0.693)	89.74	34.62	57.85	77.14	1.37	3.38
**GCs stage III vs. healthy controls**							
TrxR	0.971 (0.946–0.997)	89.13	96.15	95.86	89.94	23.17	8.85
CEA	0.923 (0.862–0.984)	84.78	100.00	100.00	86.79	−	6.57
CA72–4	0.816 (0.740–0.891)	63.04	93.85	91.11	71.15	10.24	2.54
CA19-9	0.721 (0.633–0.810)	41.30	100.00	100.00	63.01	−	1.70
**GCs stage IV vs. healthy controls**							
TrxR	0.974 (0.942–1.000)	90.48	97.69	97.51	91.12	39.21	10.26
CEA	0.912 (0.834–0.990)	76.19	100.00	100.00	80.77	−	4.20
CA72-4	0.842 (0.741–0.944)	66.67	93.85	91.55	73.79	10.83	2.82
CA19-9	0.814 (0.711–0.916)	53.68	100.00	100.00	67.74	−	2.10

Abbreviations: SEN: sensitivity; SPE: specificity; PPV: positive predictive value; NPV: negative predictive value; PLR: positive likelihood ratio; NLR: negative likelihood ratio.

### Plasma TrxR activity in gastric cancer patients with different pathological TNM stage

Previous literature has reported that the existing GC biomarkers, such as CEA, exhibited limited clinical application due to their low sensitivity in patients with early-stage GC^12,13^. In the present study, levels of TrxR, CEA, CA19-9 and CA72-4 were measured in GC patients with different TNM stage and compared with those in healthy controls (Fig. 2 and Supplemental Fig. S2). Consistent with previous publication, levels of CEA, CA72-4 and CA19-9 remained slightly altered in phase I/II GC patients compared with those in healthy controls (Fig. 2D–F and Table 2); however, plasma TrxR activity in phase I/II GC patients were significantly higher than that in healthy controls (P < 0.0001), suggesting its high sensitivity and diagnostic efficiency (AUC > 0.900) in early-stage GC diagnosis (Fig. 2C). Levels of CEA, CA72-4 and CA19-9 displayed strong elevation in phase III/IV GC patients compared with those in phase I/II GC patients (Fig. 2D–F), suggesting the diagnostic efficiency of CEA, CA19-9 and CA72-4 was remarkably improved in late-stage GC.

### Plasma TrxR activity in gastric cancer patients after chemotherapy

To further explore the efficacy of TrxR activity in evaluating the clinical outcome of GC patients after chemotherapy, a total of 662 GC patients were divided into two groups according to the clinical outcome: Clinical Responsive Patients (CRP, 456 cases) or Clinical Unresponsive Patients (CUP, 206 cases) based on their CT results. Patients with complete response (CR), partial response (PR) or stable disease (SD) mostly benefited from the chemotherapy and were included into CRP group. On the contrary, patients with progressive disease (PD) or uncontrolled condition after chemotherapy were included into CUP group. Plasma TrxR activity of both CRP and CUP groups were measured for further statistical analyses.

**Figure 3:**
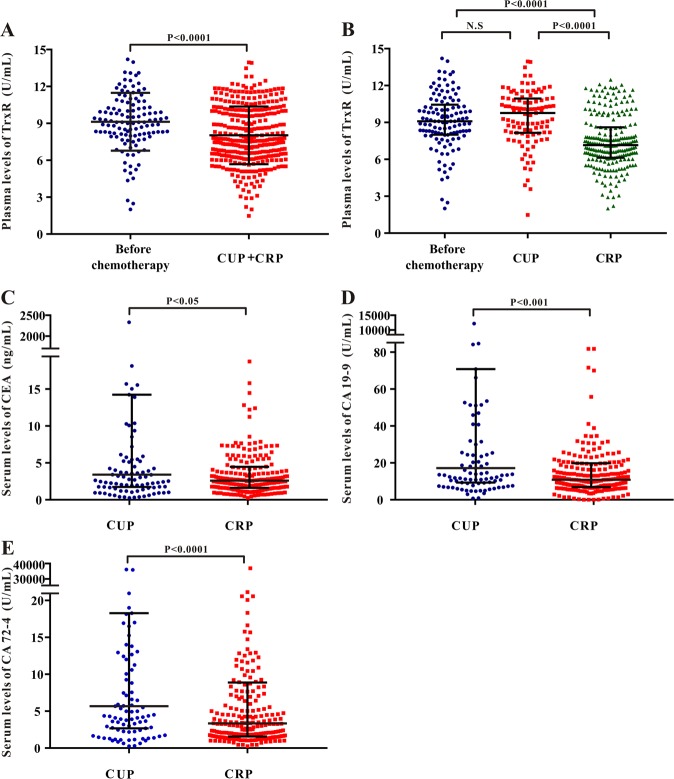
(**A**) Scatter plot of the distribution of plasma TR activity levels between GC patients before clinical interventions and GC patients after chemotherapy. (**B–E**) Scatter plot of the distribution of plasma TrxR (**B**), serum CEA (**C**), serum CA19-9 (**D**), and serum CA72-4 (**E**) among GC patients with different clinical outcome after chemotherapy (CUP vs. CRP). CUP: clinical unresponsive patient; CRP: clinical responsive patient. The black horizontal lines are median values and interquartile ranges. P values were determined by the Mann–Whitney U test. N.S: no statistical significance.

As shown in Fig. 3A, overall levels of TrxR activity in GC patients after chemotherapy [7.58 (6.26, 10.00) U/mL] was relatively lower than that in patients before clinical interventions [9.09 (7.96, 10.45) U/mL]. Notably, among the GC patients after chemotherapy, TrxR activity in CRP group [7.12 (6.08, 8.37) U/mL] was significantly lower than that in CUP group [10.07 (8.19, 11.02) U/mL], suggesting that TrxR activity was markedly reduced when patients benefit from chemotherapy (Fig. 3B). However, TrxR levels remain unaltered in CUP group in comparison with the patients before clinical interventions (P > 0.05). In consistent with TrxR activity, levels of CEA, CA72-4, and CA19-9 were also decreased in CRP group compared with CUP group (Fig. 3C–E).

### ROC analysis and cut-off value of TrxR, CEA, CA72-4, and CA19-9 in the evaluation of therapeutic efficiencies of GC after chemotherapy

**Figure 4:**
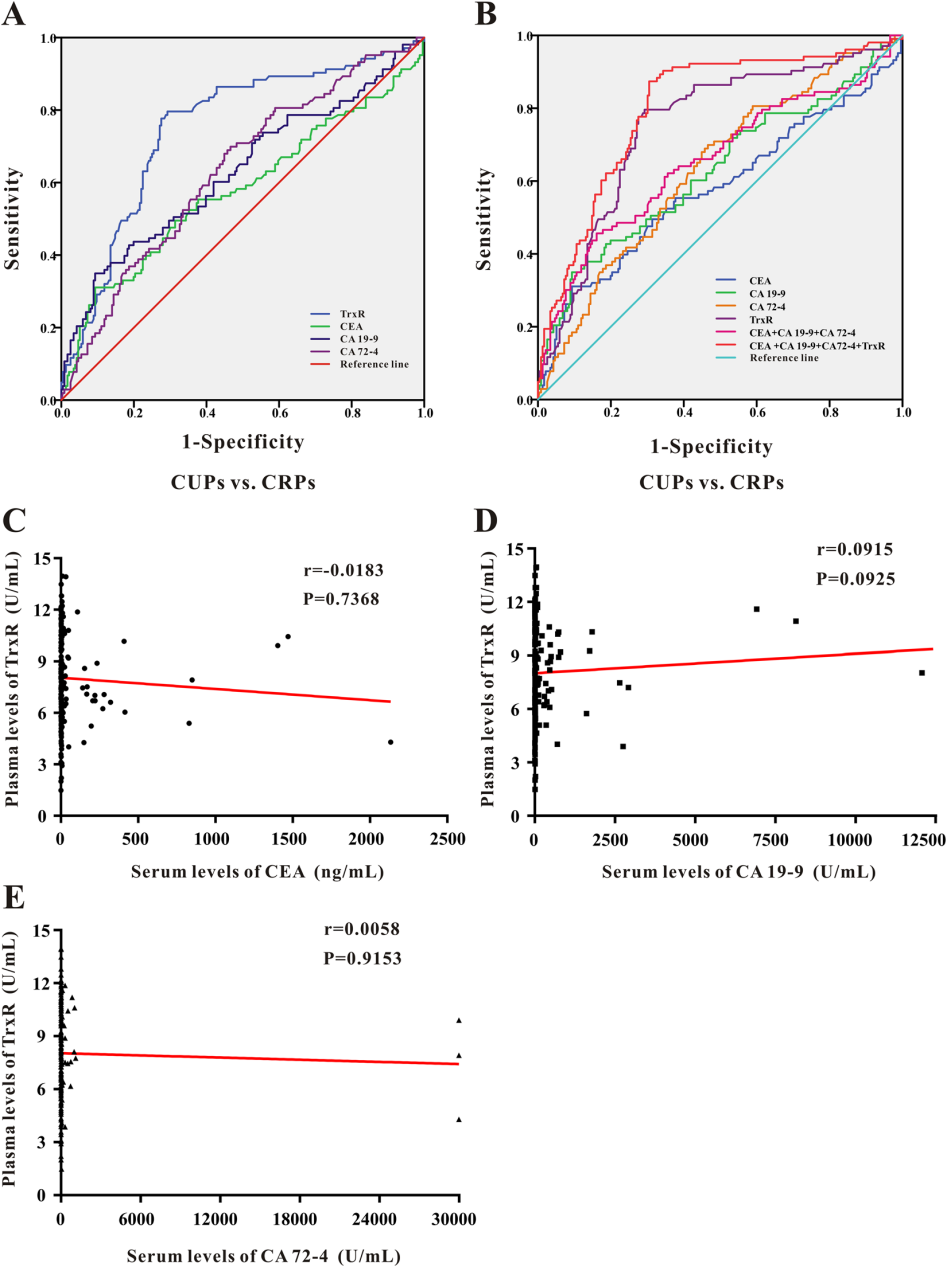
(**A,B**) ROC curve analyses of TrxR, CEA, CA19-9, CA72-4 (**A**), and the combinations thereof (**B**) for the differentiation of GCs with different clinical outcome after chemotherapy (CUPs vs. CRPs). (**C–E**) Pearson correlation analyses between TrxR activity with CEA (**C**), CA19-9 (**D**), CA72-4 (**E**) levels in GC patients after chemotherapy. P values were determined by the Spearman test.

ROC analysis was further applied to evaluate the therapeutic efficiencies of TrxR activity as well as existing GC tumor biomarkers CEA, CA72-4, CA19-9 to differentiate between CRP and CUP group (Fig. 4). The sensitivity, specificity, predictive value, and likelihood ratios of each marker are summarized in Table 3.

**Table 3:** The diagnostic efficiency of TrxR, CEA, CA72-4, CA19-9 and combinations thereof in differentiating between CUPs and CRPs in GC patients after chemotherapy.

Tumor markers	GC patients after chemotherapy						
	AUC (95%CI)	SEN%	SPE%	PPV%	NPV%	PLR	NLR
**CUPs vs. CRPs**							
TrxR	0.751 (0.693–0.808)	79.61	70.06	73.14	77.63	2.72	3.47
CEA	0.578 (0.506–0.650)	31.07	90.68	76.92	56.81	3.33	1.32
CA19-9	0.628 (0.560–0.697)	34.95	90.68	78.94	58.23	3.75	1.39
CA72-4	0.633 (0.569–0.697)	69.90	53.39	60.00	63.95	1.50	1.77
CEA + CA19-9 + CA72–4	0.655 (0.587–0.723)	45.63	83.90	73.92	60.68	2.83	1.54
CEA + CA19-9 + CA72–4 + TrxR	0.802 (0.750–0.853)	87.38	69.49	74.12	84.63	2.86	5.51

Abbreviations: CUP: clinical unresponsive patient; CRP: clinical responsive patient; SEN: sensitivity; SPE: specificity; PPV: positive predictive value; NPV: negative predictive value; PLR: positive likelihood ratio; NLR: negative likelihood ratio.

The critical cut-off value of TrxR activity levels was 7.85 U/mL.

Among four GC biomarkers, plasma TrxR activity exhibited the highest efficiency to distinguish CRPs from CUPs (AUC 0.751; 95% CI, 0.693–0.808) with a sensitivity of 79.61% and a specificity of 70.06% (Fig. 4A and Table 3). The optimal cut-off value of TrxR activity based on ROC analysis was set at 7.85 U/mL. Meanwhile, CEA, CA19-9 and CA72-4 exhibited similar capacities for differentiating CRPs from CUPs with AUCs of 0.578 (95%CI, 0.506–0.650), 0.628 (95% CI, 0.560–0.697), 0.633 (95% CI, 0.569–0.697), respectively (Fig. 4A and Table 3). These results collectively suggested TrxR as a better biomarker to evaluate the therapeutic efficiencies of GC after chemotherapy in comparison with CEA, CA19-9, and CA72-4.

It is noteworthy that the optimal cut-off values of these GC biomarkers based on ROC analyses were distinct from the thresholds recommended by the CSCO clinical guideline. According to the previous publication and CSCO clinical guideline^43,44,50–52^, the cut-off value for these GC markers were recommended at 8 U/mL for TrxR, 3.5 ng/mL for CEA, 39 U/mL for CA19-9, and 6.9 U/mL for CA72-4. Therefore, the sensitivity and specificity of these tumor markers based on the recommended cut-off values were also analyzed to evaluate the therapeutic efficiencies in GC after chemotherapy. As shown in Supplemental Fig. S3, among the four GC biomarkers, TrxR activity displayed the highest sensitivity (81.6%), and effectively distinguish CRP group from CUP group. Nevertheless, CEA, CA72-4 and CA19-9 only exhibited moderate capacities with <52% sensitivity (Supplemental Fig. S3). All these findings suggest TrxR activity as a better biomarker to monitor the therapeutic efficiencies in GC.

### Combination of TrxR, CEA, CA19-9, and CA72-4 increase the value of therapeutic evaluation in GC after chemotherapy

Levels of CEA, CA19-9 and CA72-4 are considered as important biomarkers associated with GC and commonly used in the clinical diagnosis^9–11^. Therefore, it is of great significance to investigate the value of combined detection of TrxR, CEA, CA19-9 and CA72-4 in the evaluation of therapeutic efficiencies in GC after chemotherapy. Through a binary logistic regression, the combination of CEA, CA19-9, and CA72-4 was found to exhibit an improved evaluation of therapeutic efficiency for GC patients (AUC 0.655; 95% CI, 0.587–0.723) relative to any individual biomarker (Fig. 4B and Table 3). In addition, when adding TrxR into this combined group, value of therapeutic evaluation was further strengthened for GC patients (AUC 0.802; 95% CI, 0.750–0.853) relative to TrxR alone or to the combination of other three biomarkers (Table 3). These results offered an excellent modality to evaluate the therapeutic efficiency in GC after chemotherapy.

### TrxR as an independent indicator for therapeutic efficiencies in GC after chemotherapy

To further demonstrate the clinical significance of TrxR activity in GC, we investigated the association between TrxR activity and other tumor biomarkers in GC patients after chemotherapy. As displayed in Fig. 4C–E, Pearson correlation analysis indicated no significant correlation between TrxR activity and CEA, CA19-9, or CA72-4 in GC patients. Similarly, no correlation was observed between TrxR activity with other GC biomarkers in CRP or CUP group (Supplemental Fig. S4). These results indicated plasma TrxR activity as an independent indicator for therapeutic efficiencies in GC, and TrxR level was not affected by other tumor biomarkers.

## Discussion

Numerous studies have demonstrated the crucial role of TrxR involved in the pathology of carcinomas^17,20,27–32,53,54^. Overexpression of TrxR has been observed in multiple types of tumor, including lung, kidney, breast, stomach and prostate, suggesting TrxR as a pan-cancer biomarker^35,36,48,52,55^. Further studies have confirmed the overexpression of TrxR in GC both *in vitro* and *in vivo*^39,40,56^. More importantly for diagnostic purposes, the secretion of TrxR into the peripheral blood has been observed and this secreted TrxR has proven to maintain remarkably higher levels in cancer patients compared with in healthy controls^57^. Additionally, the robust elevation of TrxR level was validated to drive the tumor growth, anti-apoptosis and activation of transcription factors in GC; while the inhibition of TrxR activity by the TrxR-specific inhibitor resulted in a strong antitumor effect in GC both i*n vitro* and *in vivo*^39,40^. All these evidences suggest that TrxR may be a novel biomarker involved in the pathology of GC.

To the best of our knowledge, it is the first study to investigate the role of TrxR in GC diagnosis and evaluation of therapeutic efficiency using clinical specimen. In this study, the detection of plasma TrxR activity in 131 GC patients before clinical intervention and 130 health controls was conducted, indicating that TrxR activity in patients [9.09 (7.96, 10.45) U/mL] was significantly higher than in health controls [3.69 (2.38, 5.32) U/mL], probably due to the elevated level of TrxR secretion under tumor growth conditions (Fig. 1A). Moreover, a ROC analysis has revealed that the diagnostic efficiency of TrxR activity in distinguishing GCs with health controls is 0.963 for AUC (95% CI, 0.943–0.983) (Fig. 2A and Table 1). Hence, the critical cut-off value of TrxR activity was set at 7.34 U/mL for GC diagnosis (SEN: 85.50%, SPE: 97.69%) based on the maximal Youden Index, which is similar to the cut-off value of plasma TrxR activity (8.0 U/mL) recommended by manufacturer and China Food and Drug Administration (CFDA). Previous studies have also reported the critical cut-off value of plasma TrxR activity in the diagnosis of multiple types of tumor, including NSCLC with 10.18 U/mL and prostate cancer with 8.20 U/mL^37,38^. All these results suggest that the threshold of TrxR activity was distinctive in the diagnosis of different tumor types.

Several tumor-specific proteins have been previously used in the clinical diagnosis of GC, such as CEA, CA72-4, and CA19-9^9–11,58^. Indeed, these proteins have been applied as biomarkers to the diagnosis and prognosis of several cancer types, including as a means of monitoring recurrence and evaluating therapeutic efficiencies in GC^59^. However, these biomarkers have exhibited limited clinical application due to their low sensitivity and diagnostic capability in patients with GC, especially in early-stage GC. Feng *et al*. have reported the low sensitivity of CEA (4.3%), CA19-9 (4.8%), AFP (1.5%), and CA125 (1.9%) in 587 cases of phase I GC patients^12^. Another study performed by Zhou *et al*. found that the sensitivity of CEA (22.4%) and CA19-9 (12.3%) was not sufficient for the diagnosis of phase I/II GC patients (1075 cases)^13^. In the present study, CEA, CA72-4, and CA19-9 only performed moderately well in GC diagnosis, and their diagnostic sensitivities (61.07% for CEA, 45.04% for CA72-4, and 29.77% for CA19-9) were relatively lower than that of plasma TrxR activity (SEN: 85.50%) (Table 1). All these findings suggest that TrxR activity appear to be a more sensitive and effective biomarker for GC diagnosis. Furthermore, we compared the diagnostic efficiency of these GC biomarkers in different stages of GC (Fig. 2C–F and Supplemental Fig. S2). Levels of CEA, CA72-4, and CA19-9 remained unchanged in phase I/II GC patients compared with those in healthy controls. On the contrary, plasma TrxR activity in phase I/II GC patients were remarkably higher than that in healthy controls, suggesting its higher sensitivity in early-stage GC diagnosis (Table 2). Interestingly, no significant change of TrxR levels was observed among different stage of GC; while levels of CEA, CA72-4 and CA19-9 displayed robust elevation in phase III/IV GC patients, indicating that the routine biomarker such as CEA may be more effective in late-stage GC diagnosis. In addition, we established a joint detection model for GC diagnosis, and incorporated CEA, CA72-4, CA19-9 and TrxR into our panel. Combination of CEA, CA72-4, and CA19-9 displayed the diagnostic sensitivity of 78.41% for GC diagnosis (Fig. 2B and Table 1). After adding TrxR into the diagnostic panel, the sensitivity greatly increased to 91.60%. Therefore, TrxR activity was well validated as an effective and efficient biomarker for GC diagnosis.

The next step was to evaluate the efficacy of TrxR activity to monitor the therapeutic efficiencies of GC patients after chemotherapy. 662 GC patients after chemotherapy were divided into CRP (Clinical Responsive Patients) and CUP (Clinical Unresponsive Patients) group according to their different clinical outcomes. Patients with complete response, partial response or stable disease mostly benefited from the chemotherapy and were included into CRP group. On the contrary, patients with progressive disease or uncontrolled condition after chemotherapy were included into CUP group. Plasma TrxR level in CRP group [7.12 (6.08, 8.37) U/mL] was remarkably decreased when compared with TrxR levels in CUP group [10.07 (8.19, 11.02) U/mL] or in GC patients before clinical interventions [9.09 (7.96, 10.45) U/mL], providing a possibility that TrxR level may be a potential indicator to monitor the therapeutic efficiencies of GC patients (Fig. 3B). Plasma TrxR level was inclined to decrease when GC patients benefited from chemotherapy, while it remained unaltered if GC patients still suffered from progressive disease after chemotherapy. Similar evidence has been previously reported in NSCLC and hepatocellular carcinoma^33,35,48^. For example, Zhou *et al*. demonstrated that almost 90% of NSCLCs patients (138/149) have shown a significant decrease of TrxR level after surgery, suggesting TrxR activity as a promising biomarker during surgery or clinical treatment^48^.

We also investigated the role of CEA, CA72-4, and CA19-9 in monitoring the therapeutic efficiencies of GC patients after chemotherapy. Although the levels of CEA, CA72-4 and CA19-9 were decreased in CRP group compared with CUP group (Fig. 3C–E), the sensitivity of these biomarkers to discriminate between CRP and CUP was still lower than that of plasma TrxR level (Fig. 4A and Table 3). Notably, using the CSCO recommended thresholds of biomarkers instead of the critical cut-off values calculated by ROC analyses, the sensitivities of CEA, CA72-4 and CA19-9 in monitoring the therapeutic efficiencies of GC patients were even declined to 51.4%, 44.6%, and 34.9%, respectively (Supplemental Fig. S3). However, TrxR level still maintained relatively high efficiency with a sensitivity of 81.6% and specificity of 66.1% (Supplemental Fig. S3A). Moreover, the combination detection panel of biomarkers was applied to evaluate the GC therapeutic efficiency. The combination of CEA, CA72-4 and CA19-9 was found to exhibit an improved evaluation of therapeutic efficiency for GC patients relative to any individual biomarker (Fig. 4B and Table 3). When adding TrxR into this combined group, diagnostic efficacy was further strengthened for GC patients relative to TrxR alone or to the combination of routine biomarker screening (Table 3). According to a Pearson correlation analysis results, no significant correlation between TrxR activity and CEA, CA19-9, or CA72-4 was identified, suggesting plasma TrxR activity as an independent indicator for therapeutic efficiencies in GC (Fig. 4C–E and Supplemental Fig. S4). Together, in the present study, all evidences indicate TrxR as a promising biomarker to monitor the therapeutic efficiency of GC patients in future clinical application.

At present, routine biomarker screening (CEA, CA72-4, and CA19-9) is the recommended approach for GC diagnosis and evaluation of therapeutic efficiency, considering the invasive diagnostic strategies such as gastroscope always lead to severe pain and inconvenience^8,60^ In this study, we proposed plasma TrxR activity as a novel, independent and efficient biomarker for GC diagnosis and therapeutic evaluation. In both cases, TrxR activity displayed higher diagnostic AUC and sensitivity when compared with CEA, CA72-4, and CA19-9. Meanwhile, the combination of TrxR activity with routine biomarkers can further improve the GC diagnostic value and offer a better diagnostic panel for GC patients. However, it is noteworthy that most participants in this retrospective study were from eastern China, and therefore, the presence of common confounding variables and comorbid conditions cannot be ignored. Given the distinct pathogeny of GC existed in different region and ethnicity, future studies are definitely necessary in different cohort of GC patients. Besides, the molecular mechanisms of TrxR activity in GC patients remain to be elucidated, which may further explain the reason of elevated TrxR levels in GC patients.

In summary, this is the first study to investigate the diagnostic relevance of TrxR activity as a plasma biomarker in GCs. Our results indicated that plasma TrxR activity is an effective, efficient and independent biomarker for GC diagnosis and evaluation of therapeutic efficiency, suggesting TrxR activity as a better diagnostic tool in future GC clinical application.

## Supplementary information

Supplementary Information
