# Author Correction: Plasma activity of Thioredoxin Reductase as a Novel Biomarker in Gastric Cancer

**DOI:** 10.1038/s41598-020-70071-5

**Published:** 2020-10-14

**Authors:** Wei Peng, Zhaofei Zhou, Yuejiao Zhong, Yan Sun, Yajing Wang, Zili Zhu, Wenxuan Jiao, Man Bai, Jing Sun, Jianwei Lu, Hanwei Yin

**Affiliations:** 1 Department of Medicine, Jiangsu Cancer Hospital & Jiangsu Institute of Cancer Research & The Affiliated Cancer Hospital of Nanjing Medical University, Nanjing, China; 2 Nantong Tumor Hospital, Nantong, China; 3 State Key Laboratory of Natural and Biomimetic Drugs, Peking University Health Science Center, Beijing, China; 4 Keaise Center for Clinical Laboratory, Wuhan, China

Correction to: *Scientific Reports* https://doi.org/10.1038/s41598-019-55641-6, published online 13 December 2019

The original version of this Article contained an error in the order of the author names, which were given incorrectly as ‘Wei Peng, Zhaofei Zhou, Yuejiao Zhong, Yan Sun, Yajing Wang, Zili Zhu, Wenxuan Jiao, Man Bai, Jing Sun, Hanwei Yin & Jianwei Lu’

This error has now been corrected in the HTML and PDF versions of this Article, and in the accompanying Supplementary Information file.

