# Supplementary material for: Plasma activity of Thioredoxin Reductase as a Novel Biomarker in Gastric Cancer
Source: Sci Rep. 2019 Dec 13;9:19084. doi: 10.1038/s41598-019-55641-6 (PMC6910980; doi:10.1038/s41598-019-55641-6)
Supplement: Supplementary file 1 — Supplementary Information [file 41598_2019_55641_MOESM1_ESM.docx]

**Plasma activity of Thioredoxin Reductase as a Novel Biomarker in Gastric Cancer**

Wei Peng^1, #^, Zhaofei Zhou^1, #^, Yuejiao Zhong^1^, Yan Sun^1^, Yajing Wang^1^, Zili Zhu^2^, Wenxuan Jiao^3^, Man Bai^3^, Jing Sun^3^, Hanwei Yin^4, *^, Jianwei Lu^1, *^

Authors Affiliations: ^1^Department of Medicine, Jiangsu Cancer Hospital & Jiangsu Institute of Cancer Research & The Affiliated Cancer Hospital of Nanjing Medical University, Nanjing, China; ^2^Nantong Tumor Hospital, Nantong, China; ^3^State Key Laboratory of Natural and Biomimetic Drugs, Peking University Health Science Center, Beijing, China; ^4^Keaise Center for Clinical Laboratory, Wuhan, China;

^#^ These authors contributed equally to this work

*Corresponding Author:

Jianwei Lu: Department of Medicine, Jiangsu Cancer Hospital & Jiangsu Institute of Cancer Research & The Affiliated Cancer Hospital of Nanjing Medical University, Nanjing 210000, China. E-mail address: lujw@medmail.com.cn.

Hanwei Yin: Keaise Center for Clinical Laboratory, Wuhan 430075, China. E-mail address: 18911420767@163.com.

**Running head:** TrxR as a novel biomarker for gastric cancer

**Conflicts of interest statement**

The authors declare no potential conflicts of interest

**Supplemental Experimental Procedures**

**Sample preparation for TrxR activity**

We followed a standard procedure to prepare the human specimens, which was also required by the manufacturer’s instruction. Samples from preoperative peripheral blood were collected in EDTA or anticoagulant-free tubes, followed by centrifugation at 3,500 rpm at room temperature for 5 minutes within 2 hours of collection. The supernatants were collected in 4°C and tested immediately. The storage temperature is 4°C, while the incubation temperature is 37°C. The low storage temperature and short handling time have made it least possible that the levels of free thiols in the plasma were altered during sample preparation or storage.

**Determination and calculation of TrxR activity**

As described in **Materials and methods**, TrxR activity was measured by commercially available thioredoxin reductase (TrxR) activity colorimetric assay kits (Clairvoyance Health Technology, China), which was based on DTNB reduction and performed according to the manufacturer’s instruction. Positive and negative controls from the kits are included in each reaction to monitor the assay performance.

The calculation of TrxR activity is described as follows:

**U/ml = ∆A412/min (thioredoxin reductase) × dil × Vol / (vol × L × ε)**

∆A412/min (thioredoxin reductase) = [∆A412/min (sample) - ∆A412/min (sample + inhibitor)]; dil = sample dilution factor; Vol = volume of reaction in ml; vol = volume of sample in ml; L = length of [optical path](http://www.baidu.com/link?url=qKlH69dtCI0DB-aFzmG_15ePnvC2oXnv-CuTmlytikvnActu4rnwBFBkjBZY2XjoO6dCnqVzRLU1C6xxM0nyXppWEbF8Uzhp5Tww-RU3BN5So2t61EGIn0F6dU6NznMD); ε = extinction coefficient; ∆A412/min (sample)= [A412 (sample, Xmin) - A412/min (sample,0min)] / Xmin; ∆A412/min (sample + inhibitor) = [A412 (sample + inhibitor, Xmin) - A412 (sample + inhibitor,0min)] / Xmin.

The absolute values of absorbances of all patient samples were analyzed by KEA-TR100 analyzer (Clairvoyance, China), and its absolute absorbance linear range at 412 nm is 0.60-3.00. Any absolute absorbance outside this limitation was considered inaccurate and excluded from the study.

**Supplemental Figure S1**


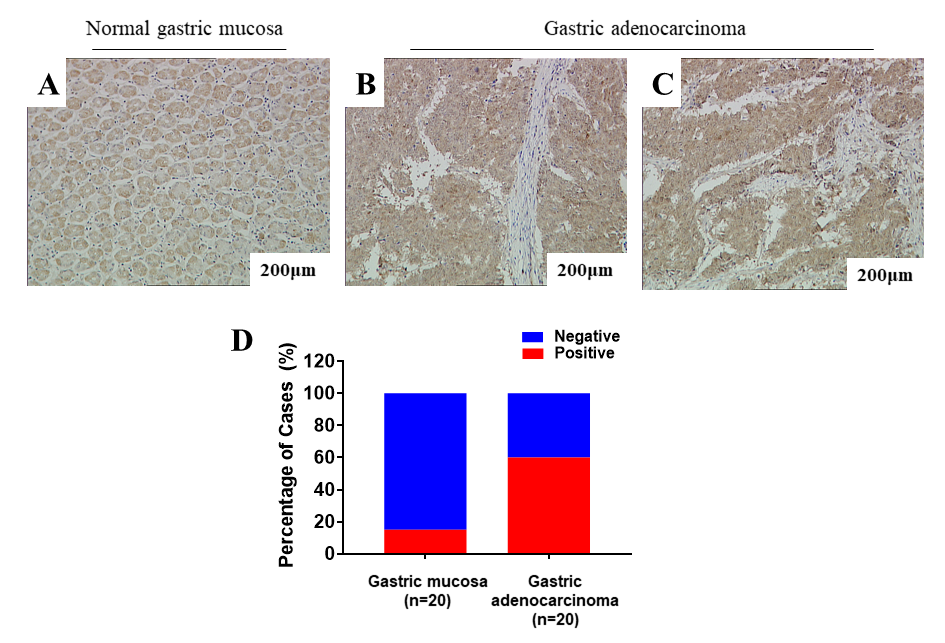


Supplemental Figure S1. TrxR expression in normal gastric mucosa and adenocarcinoma tissues. (A-C) Representative IHC staining of TrxR in normal gastric mucosa tissue (A) and gastric adenocarcinoma tissues (B and C). (D) TrxR expression rates in normal gastric mucosa and adenocarcinoma tissues: TrxR expression was positive (+) in 60% (12/20) of the gastric adenocarcinoma tissues.

**Supplemental Figure S2**


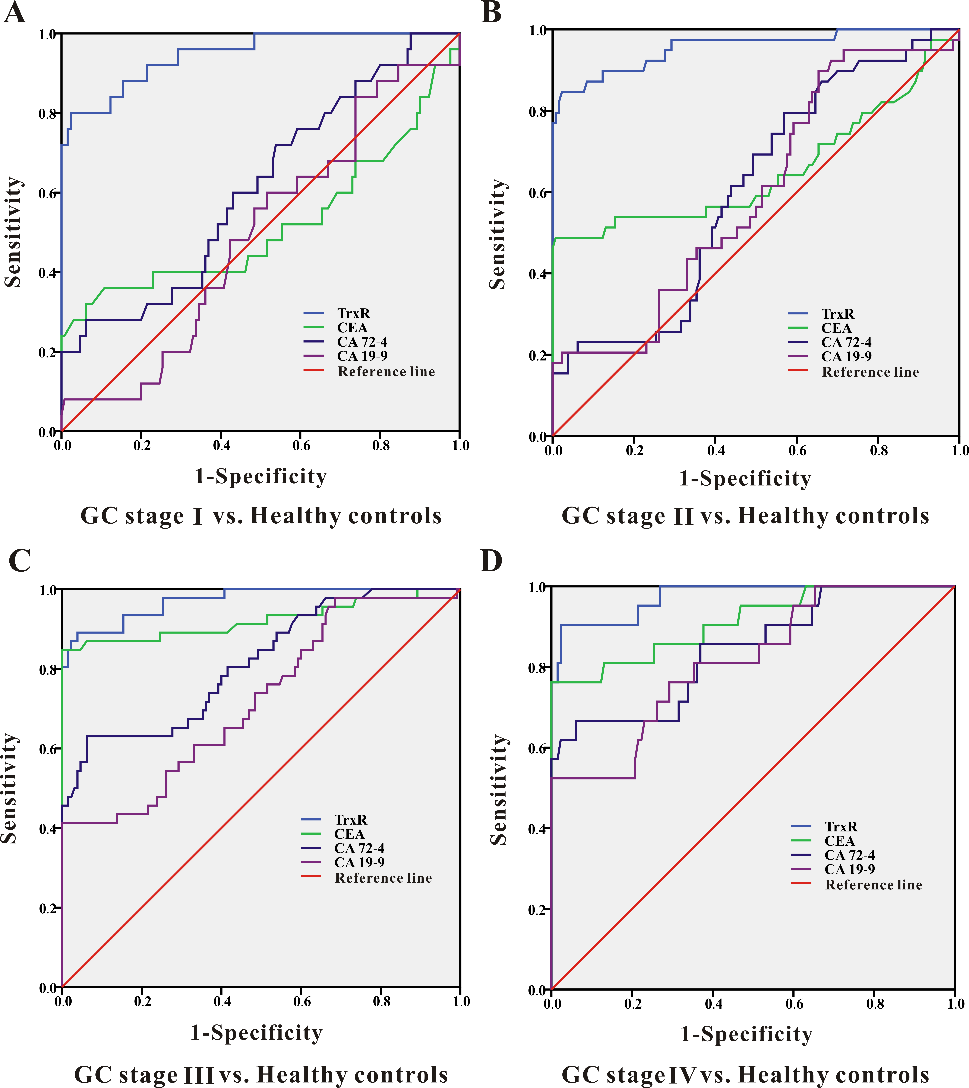


Supplemental Figure S2. (A-D) ROC curve analyses of TrxR, CEA, CA19-9, and CA72-4 levels for the differentiation of healthy controls and GC patients with various pathological TNM stages.

**Supplemental Figure S3**


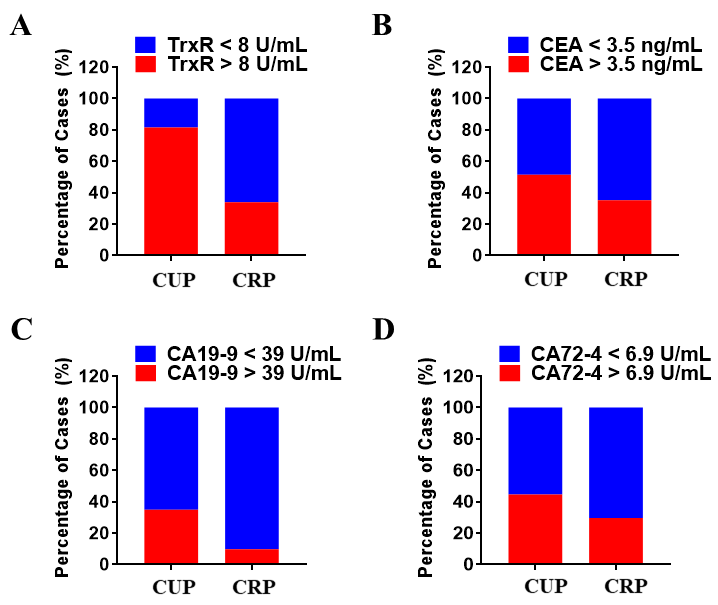


Supplemental Figure S3. (A-D) Sensitivity and specificity of TrxR (SEN:81.55%, SPE:66.10%), CEA (SEN:51.46%, SPE:64.83%), CA19-9 (SEN:34.95%, SPE:90.25%), and CA72-4 (SEN:44.66%, SPE:70.34%) levels based on recommended cut-off values in GC patients with different clinical outcome after chemotherapy (CUP vs. CRP). CUP: clinical unresponsive patient; CRP: clinical responsive patient.

**Supplemental Figure S4**


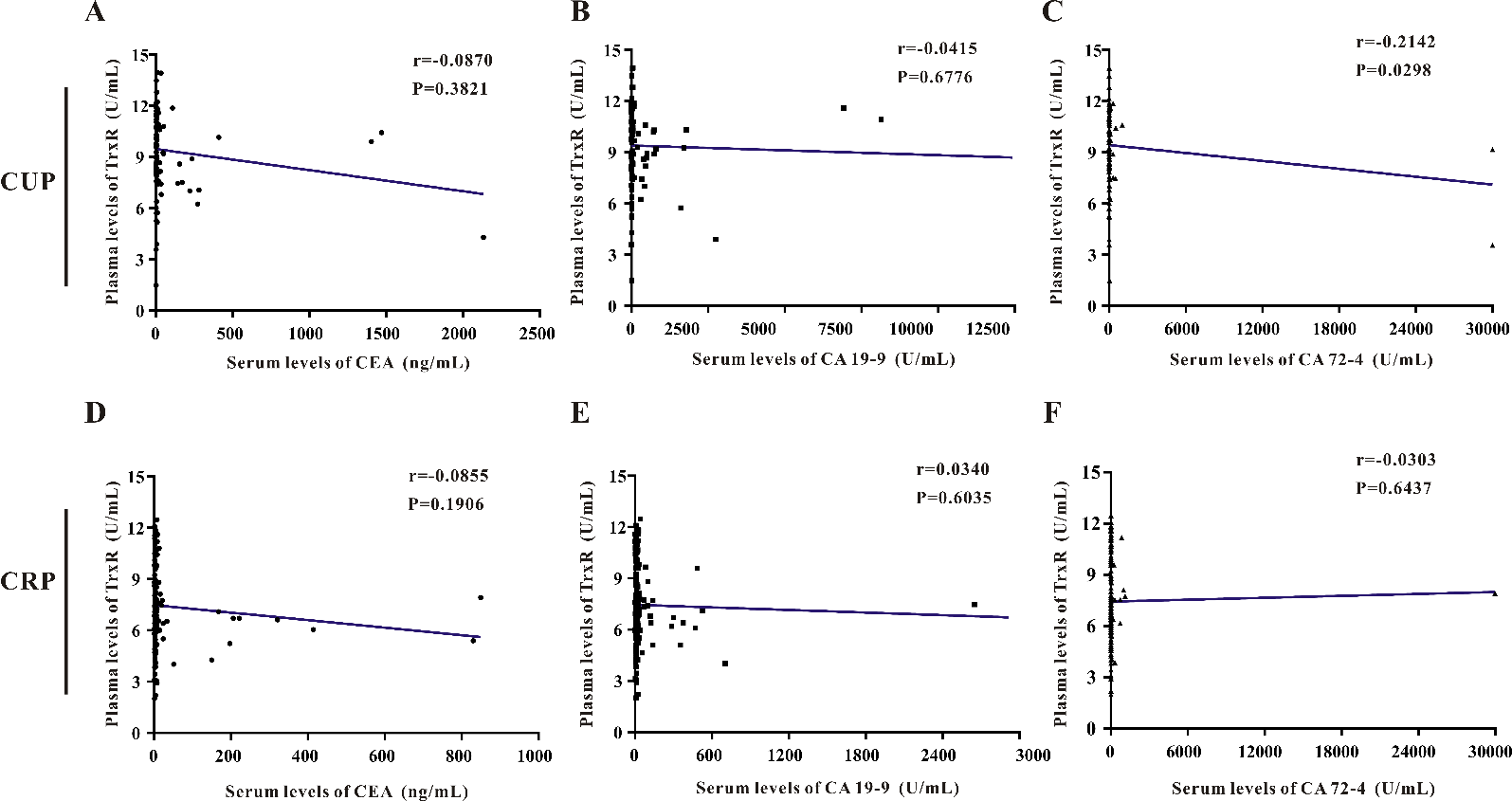


Supplemental Figure S4. (A-C) Pearson correlation analyses between TrxR activity with CEA (A), CA19-9 (B), CA72-4 (C) levels in CUPs after chemotherapy. (D-F) Pearson correlation analyses between TrxR activity with CEA (D), CA19-9 (E), CA72-4 (F) levels in CRPs after chemotherapy. P values were determined by the Spearman test.

**Supplemental Table S1**

| **Characteristics** | **Gastric cancer** | | **Healthy controls** |
| --- | --- | --- | --- |
|  | **Before clinical interventions** | **After chemotherapy** |  |
| N | 131 | 662 | 130 |
| Age（IQR,years） | 64 (51-70) | 63 (55-70) | 58 (43-70) |
| **Gender**（**%**） | | | |
| Male | 88 (67.2) | 426 (64.4) | 72 (55.4) |
| Female | 43 (32.8) | 236 (35.6) | 58 (44.6) |
| **Histologiacal type**（**%**） | | | |
| Adenocarcinoma | 128 (97.7) | 639 (96.5) | — |
| Others | 3 (2.2) | 23 (3.5) | — |
| **TNM**（**%**） | | | |
| Ⅰ | 25 (19.1) | 40 (6.0) | — |
| Ⅱ | 39 (29.8) | 148 (22.4) | — |
| Ⅲ | 46 (35.1) | 179 (27.0) | — |
| Ⅳ | 21 (16.0) | 295 (44.6) | — |
| **Distant metastasis**（**%**） | | | |
| No | 110 (84.0) | 367 (55.4) | — |
| Yes | 21 (16.0) | 295 (44.6) | — |

Supplemental Table S1. The characteristics of gastric cancer and healthy control cases in the study.
